# Polymorphisms in *TIE2* and *ANGPT-1* genes are associated with protection against diabetic retinopathy in a Brazilian population

**DOI:** 10.20945/2359-3997000000624

**Published:** 2023-05-29

**Authors:** Cristine Dieter, Natália Emerim Lemos, Nathalia Rodrigues de Faria Corrêa, Taís Silveira Assmann, Felipe Mateus Pellenz, Luís Henrique Canani, Letícia de Almeida Brondani, Andrea Carla Bauer, Daisy Crispim

**Affiliations:** 1 Hospital de Clínicas de Porto Alegre Porto Alegre RS Brasil Hospital de Clínicas de Porto Alegre, Serviço de Endocrinologia, Porto Alegre, RS, Brasil; 2 Universidade Federal do Rio Grande do Sul Faculdade de Medicina Departamento de Clínica Médica Porto Alegre RS Brasil Universidade Federal do Rio Grande do Sul, Faculdade de Medicina, Departamento de Clínica Médica, Programa de Pós-graduação em Ciências Médicas: Endocrinologia, Porto Alegre, RS, Brasil; 3 Hospital de Clínicas de Porto Alegre Divisão de Nefrologia Porto Alegre RS Brasil Hospital de Clínicas de Porto Alegre, Divisão de Nefrologia, Porto Alegre, RS, Brasil

**Keywords:** *ANGPT-1* gene, *TIE2* gene, polymorphism, type 2 diabetes mellitus, diabetic retinopathy

## Abstract

**Objective:**

The objective of this study was to investigate the association between SNPs in the *TIE2* and *ANGPT-1* genes and diabetic retinopathy (DR).

**Subjects and methods:**

This study comprised 603 patients with type 2 diabetes mellitus (T2DM) and DR (cases) and 388 patients with T2DM for more than 10 years and without DR (controls). The *TIE2* rs639225 (A/G) and rs638203 (A/G) SNPs and the *ANGPT-1* rs4324901 (G/T) and rs2507800 (T/A) SNPs were genotyped by real-time PCR using TaqMan MGB probes.

**Results:**

The G/G genotype of the rs639225/*TIE2*, the G/G genotype of the rs638203/*TIE2* and the T allele of the rs4324901/*ANGPT-1* SNPs were associated with protection against DR after adjustment for age, glycated hemoglobin, gender, and presence of hypertension (P = 0.042, P = 0.003, and P = 0.028, respectively). No association was found between the rs2507800/*ANGPT-1* SNP and DR.

**Conclusion:**

We demonstrated, for the first time, the association of *TIE2* rs638203 and rsrs939225 SNPs and *ANGPT-1* rs4324901 SNP with protection against DR in a Brazilian population.

## INTRODUCTION

Diabetic retinopathy (DR) is one of the commonest complications of diabetes mellitus (DM) and is characterized by damage to small blood vessels of the retina (1). DR is a neurovascular disorder and the leading cause of legal blindness in working-age adults, affecting over 93 million people worldwide (2, 3). Clinically, DR is classified into two main stages: nonproliferative diabetic retinopathy (NPDR) and proliferative diabetic retinopathy (PDR). NPDR is the first stage of DR, characterized by increased vascular permeability, capillary occlusion, microaneurysms, bleeding, and hard exudates (4). In contrast, PDR is a progressive stage of DR characterized by neovascularization (4).

Vascular growth and angiogenesis are involved in the pathogenesis of this diabetic complication. Angiopoietins (ANGPTs) are key regulators of these processes. ANGPT-1 is involved in vascular maturation, endothelial cell (EC) survival, EC interactions with other cells, and vascular permeability (5). In contrast, ANGPT-2 is an ANGPT-1 antagonist and inhibits endothelial quiescence, neutralizing the vascular maintenance activities of ANGPT-1 in cases where endothelial remodeling is necessary, such as during inflammation and angiogenesis (6, 7).

ANGPT-1 and ANGPT-2 signaling occur through transmembrane tyrosine kinase receptor (TIE2) (6-8), which is expressed in both vascular endothelial tissue and non-EC, and has a vital role in vascular stability (9-13). ANGPT-1 activation by TIE2 generally leads to protective effects on cells, such as cell migration, adhesion, and survival. ANGPT-2 acts as a partial agonist of TIE2, where its high concentrations lead to a competitive inhibition of ANGPT-1 signaling through TIE2 (14).

In this context, studies have reported the involvement of ANGPT-1 and TIE2 in the pathogenesis of chronic complications of DM (15). Khalaf and cols. (16) reported that serum ANGPT-1 levels were higher in patients with NPDR compared to patients with DM but without DR, while no differences were found between the PDR group and control patients with DM. Serum TIE2 levels did not differ between patients with and without DR (16). Another study showed that ANGPT-1 and ANGPT-2 levels were increased in vitreous samples of patients with PDR compared to control patients without DM (17). Moreover, Jeansson and cols. (18) demonstrated that diabetic mice with *Angpt-1* deletion and streptozotocin-induced diabetes developed more severe diabetic kidney disease (DKD) compared to diabetic mice with normal *Angpt-1* expression. Taking this background into consideration, single nucleotide polymorphisms (SNPs) that could influence the expression of these genes may be associated with susceptibility to chronic complications of DM, including DR. Therefore, we investigated, for the first time, the association of rs639225 (A/G) and rs638203 (A/G) SNPs in the *TIE2* gene and rs4324901 (G/T) and rs2507800 (T/A) SNPs in the *ANGPT-1* gene with DR.

## SUBJECTS AND METHODS

### Study participants

Strengthening the Reporting of Observational Studies in Epidemiology (STROBE) and Strengthening the Reporting of Genetic Association Studies (STREGA) guidelines were used to design this case-control study (19, 20). The sample consisted of 991 unrelated patients with type 2 DM (T2DM) selected from *Hospital de Clínicas de Porto Alegre* and *Grupo Hospitalar Conceição* (Porto Alegre, Rio Grande do Sul, Brazil) between 2002 and 2013, as previously described (21).

T2DM was diagnosed according to American Diabetes Association criteria (22). The diagnosis of DR was made by an experienced ophthalmologist using fundoscopy through dilated pupils. DR was classified as “absent DR” (no fundus abnormalities), ‘NPDR’ (microaneurysms, hemorrhage, and hard exudates), or “PDR” (newly formed blood vessels and/or growth of fibrous tissue into the vitreous cavity). DR classification considered the most severely affected eye, according to the scale developed by the Global Diabetic Retinopathy Group (23). Cases were defined by the presence of DR (NPDR or PDR). Controls were defined by the absence of this complication and known DM for at least 10 years.

A standard questionnaire was used to collect information about age, age at T2DM diagnosis, T2DM duration, and pharmaceutical treatment, and all patients underwent physical and laboratory evaluations as previously described (24). Serum and plasma samples were taken after 12 h of fasting for laboratory analyses (24). Glycated hemoglobin (HbA1c) measurements were performed by different methods and the results were traceable to the Diabetes Control and Complications Trial (DCCT) method by off-line calibration or through conversion formulae (25). Creatinine was measured by the Jaffe reaction; total plasma cholesterol, high-density lipoprotein (HDL) cholesterol, and triglycerides were analyzed by enzymatic methods, and albuminuria was verified by immunoturbidimetry (Sera-Pak Immuno Microalbumin kit, Bayer, Tarrytown, NY, USA; mean intra- and inter-assay coefficients of variance of 4.5% and 11%, respectively) (26).

DKD was diagnosed based on the Kidney Disease Improving Global Outcomes (KDIGO) guidelines (27), using both urinary albumin excretion (UAE) levels and estimated glomerular filtration rate (eGFR); the latter was calculated using the Chronic Kidney Disease Epidemiology Collaboration (CKD-EPI) equation (28). Ethnic groups were defined based on self-classification.

The study protocol was approved by the Ethic Committee in Research from *Hospital de Clínicas de Porto Alegre* (CAAE number: 97844918.1.0000.5327), and all participants provided assent and written informed consent prior to inclusion in the study.

### Genotyping

DNA was extracted from peripheral blood leucocytes using a standardized salting-out procedure (29). The rs639225 (A/G) (Assay ID = C__1305224_30) and rs638203 (A/G) (Assay ID = C__8775841_10) SNPs in the *TIE2* gene, as well as the rs4324901 (G/T) (Assay ID = C__26472342_10) and rs2507800 (T/A) (Assay ID = C__1252396_10) SNPs in the *ANGPT-1* gene were genotyped using specific Human TaqMan SNP Genotyping Assays 40x (Thermo Fisher Scientific, Foster City, CA, USA). Real-time PCR reactions were performed in 384-well plates, in a volume of 5 µL, using 2 ng of DNA, TaqPath ProAmp 1 X Mastermix (Thermo Fischer Scientific), and TaqMan SNP Genotyping Assay 1 X. Plates were placed in a real-time PCR thermal cycler (ViiA7 Real-Time PCR System; Thermo Fisher Scientific) and heated for 10 min at 95 °C, followed by 50 cycles of 95 °C for 15 s and 62 °C for 1 min.

### Statistical analyses

Allele frequencies were determined by gene counting, and departures from Hardy–Weinberg equilibrium (HWE) were verified using the *x*^2^ test. Allele and genotype frequencies were compared between groups using *x*^2^ tests. Genotypes were also compared between groups under additive, recessive, and dominant inheritance models, categorized as suggested by a previous publication (30). We also examined the widely used measures of linkage disequilibrium (LD), Lewontin's D’ (|D’|), and r^2^ between all pairs of biallelic loci (31). Haplotypes constructed with the combination of the two *TIE2* and two *ANGPT-1* SNPs and their frequencies were inferred using PHASE 2.1 software, which implements a Bayesian statistical method (32).

Clinical and laboratory characteristics were compared between groups of patients categorized according to the different SNP genotypes using an unpaired Student's t-test or a *x*^2^ test, as appropriate. Normal distribution of quantitative variables was checked using Kolmogorov-Smirnov and Shapiro–Wilk tests. Variables with normal distribution are shown as means ± standard deviation (SD). Variables with skewed distribution were log-transformed before the analyses and are shown as medians (25th-75th percentile values). Categorical variables are shown as n (%). Multivariate logistic regression analyses were done to evaluate the independent association of SNPs with DR, adjusting for possible confounding factors. Variables with significant associations with DR in the univariate analysis or with a biologically relevant association with this complication were chosen for inclusion in the multivariate model. DM duration was not included as an independent variable in these analyses since the DM control group was selected based on this characteristic. Statistical analyses were performed using SPSS 18.0 software (SPSS, Chicago, IL), and P values < 0.05 were considered significant.

Sample sizes were calculated using the OpenEpi website (www.openepi.com). Since no previous study investigated the association of the SNPs of interest in *TIE2* and *ANGPT-1* genes with DR, data from studies that evaluated the association of these SNPs with other diseases were used (mean minor allele frequency = 0.30 and odds ratio [OR] = 0.4 or 1.4) (33-35). Therefore, the calculated sample size was estimated at 403 controls and 504 cases to find an OR = 1.4, with 80% power; or 113 controls and 142 cases to find an OR = 0.4, with 80% power.

## RESULTS

### Sample description

Table 1 describes the clinical and laboratorial characteristics of patients with T2DM for more than 10 years without DR (controls) and patients with T2DM and DR (cases). As expected, mean HbA1c and UAE levels, as well as the prevalence of arterial hypertension and DKD, were higher in patients with T2DM and DR compared to controls (all P values < 0.050). Moreover, the frequency of males and non-white subjects and LDL cholesterol levels were increased in the case group compared to controls (all P values < 0.050). Mean age, BMI, and eGFR levels were lower in cases compared to controls (all P values < 0.050).

**Table 1:** Clinical and laboratory characteristics of patients with DM (controls) and patients with DM and DR (cases)

Characteristics	Patients with T2DM		P *
	Without DR n = 388	With DR n = 603	
Age (years)	69.4 ± 10.5	65.8 ± 10.5	0.0001
Gender (% male)	37.4	51.6	0.0001
BMI (kg/m²)	29.0 ± 5.1	28.2 ± 5.2	0.017
Ethnicity (% non-white)	18.4	25.2	0.017
HbA1c (%)	7.4 ± 1.8	7.8 ± 2.1	0.005
Hypertension (%)	82.3	91.2	0.0001
Duration of diabetes (years)	20.6 ± 8.8	20.6 ± 9.6	0.935
Cholesterol total (mg/dL)	194.9 ± 49.6	198.3 ± 51.3	0.340
Triglycerides (mg/dL)	148.0 (103.5-209.0)	149.0 (102.0-215.0)	0.818
HDL cholesterol (mg/dL)	46.5 ± 12.6	45.3 ± 14.0	0.163
LDL cholesterol (mg/dL)	112.7 ± 43.5	119.2 ± 46.4	0.045
eGFR (mL/min per 1.73 m^2^)	74.0 (51.0-90.0)	53.0 (16.0-76.0)	0.0001
UAE (mg/g)	8.3 (3.1-36.6)	53.1 (8.6-369.6)	0.0001
Diabetic kidney disease (%)	48.4	75.7	0.0001

Variables are shown as means ± standard deviations (SD), median (25th-75th percentiles), or %.

* P value was computed using Student's t-tests or *x*^2^ tests, as appropriate. T2DM: type 2 diabetes mellitus; BMI: body mass index; DR: diabetic retinopathy; HDL: high-density lipoprotein; LDL: low-density lipoprotein; eGFR: estimated glomerular filtration rate; HbA1c: glycated hemoglobin; T2DM: type 2 diabetes mellitus; UAE: urinary albumin excretion.

### Association between SNPs in *TIE2* and *ANGPT-1* genes and DR

Table 2 shows genotype and allele frequencies of rs639225 and rs638203 in *TIE2* gene and of rs4324901 and rs2507800 in *ANGPT-1* gene between patients with T2DM without DR (controls) and patients with T2DM and DR (cases). The allele and genotype frequencies of the *TIE2* rs639225 SNP did not differ significantly between groups (P = 0.107 and P = 0.211, respectively). However, after adjusting for age, HbA1c, gender, and presence of hypertension, the rs639225 G/G genotype was associated with protection against DR (OR = 0.642, 95% CI 0.419-0.984; P = 0.042). This association was also found when considering the additive inheritance model (OR = 0.643, 95% CI 0.419-0.986; P = 0.043).

**Table 2:** Genotype and allele frequencies of SNPs in the *TIE2* and *ANGPT-1* genes in patients with T2DM, with and without DR

	Patients with DM (controls)	Patients with DR (cases)	Unadjusted P*	Adjusted OR (95% CI) /† P
***rs639225 – TIE2***	368	547		
**Genotype**				
A/A	119 (32.3)	193 (35.3)		1
A/G	157 (42.7)	244 (44.6)		0.886 (0.622-1.264)/0.505
G/G	92 (25.0)	110 (20.1)	0.211	0.642 (0.419-0.984)/0.042
**Allele**				
A	0.54	0.58		
G	0.46	0.42	0.107	
**Recessive model**				
A/A + A/G	276 (75.0)	437 (79.9)		1
G/G	92 (25.0)	110 (20.1)	0.095	0.687 (0.471-1.001)/0.051
**Additive model**				
A/A	119 (56.4)	193 (63.7)		1
G/G	92 (43.6)	110 (36.3)	0.115	0.643 (0.419-0.986)/0.043
**Dominant model**				
A/A	119 (32.3)	193 (35.3)		1
A/G + G/G	249 (67.7)	354 (64.7)	0.395	0.798 (0.575-1.108)/0.178
**rs638203 – *TIE2***	368	562		
**Genotype**				
A/A	118 (32.1)	197 (35.1)		1
A/G	156 (42.4)	260 (46.3)		0.921 (0.645-1.313)/0.648
G/G	94 (25.5)	105 (18.7)	0.046	0.523 (0.340-0.804)/0.003
**Allele**				
A	0.53	0.58		
G	0.47	0.42	0.040	
**Recessive model**				
A/A + A/G	274 (74.5)	457 (81.3)		1
G/G	94 (25.5)	105 (18.7)	0.016	0.548 (0.376-0.800)/0.002
**Additive model**				
A/A	118 (55.7)	197 (65.2)		1
G/G	94 (44.3)	105 (34.8)	0.036	0.527 (0.343-0.812)/0.004
**Dominant model**				
A/A	118 (32.1)	197 (35.1)		1
A/G + G/G	250 (67.9)	365 (64.9)	0.384	0.770 (0.554-1.070)/0.119
**rs4324901 – *ANGPT-1***	354	532		
**Genotype**				
G/G	152 (42.9)	271 (50.9)		1
G/T	149 (42.1)	194 (36.5)		0.724 (0.514-1.019)/0.064
T/T	53 (15.0)	67 (12.6)	0.064	0.640 (0396-1.034)/0.068
**Allele**				
G	0.64	0.69		
T	0.36	0.31	0.026	
**Recessive model**				
G/G + G/T	301 (85.0)	465 (87.4)		1
T/T	53(15.0)	67(12.6)	0.361	0.742 (0.473-1.166)/0.196
**Additive model**				
G/G	152 (74.1)	271 (80.2)		1
T/T	53 (25.9)	67 (19.8)	0.125	0.651 (0.402-1.053)/0.080
**Dominant model**				
G/G	152 (42.9)	271 (50.9)		1
G/T + T/T	202 (57.1)	261 (49.1)	0.023	0.701 (0.510-0.963)/0.028
**rs2507800 – *ANGPT-1***	356	547		
**Genotype**				
T/T	154 (43.3)	246 (45.0)		1
T/A	142 (39.9)	221 (40.4)		0.1030 (0.733-1.448)/0.865
A/A	60 (16.9)	80 (14.6)	0.656	0.832 (0.523-1.323)/0.437
**Allele**				
T	0.63	0.65		
A	0.37	0.35	0.421	
**Recessive model**				
T/T + T/A	296 (83.1)	467 (85.4)		1
A/A	60 (16.9)	80 (14.6)	0.418	0.820 (0.531-1.267)/0.371
**Additive model**				
T/T	154 (72.0)	246 (75.5)		1
A/A	60 (28.0)	80 (24.5)	0.420	0.835 (0.523-1.333)/0.449
**Dominant model**				
T/T	154 (43.3)	246 (45.0)		1
T/A + A/A	202 (56.7)	301 (55.0)	0.661	0.972 (0.709-1.333)/0.861
**Haplotype *ANGPT-1***	331	494		
0-2 mutated alleles	262 (79.2)	418 (84.6)		1
3-4 mutated alleles	69 (20.8)	76 (15.4)	0.054	0.602 (0.392-0.925)/0.021

Data are shown as number (%) or proportion.

* P-values were calculated using *x*^2^ tests.

† P-values and OR (95% CI) obtained using logistic regression analyses adjusting for age, gender, HbA1c, and presence of hypertension. DM: diabetes mellitus; DR: diabetic retinopathy; OR: odds ratio; CI: confidence interval.

Frequencies of the *TIE2* rs638203 G allele and G/G genotype were higher in controls compared to cases (P = 0.040 and P = 0.046, respectively). After adjustment for the same covariates described above, the G/G genotype was associated with protection against DR (OR = 0.523, 95% CI 0.340-0.804; P = 0.003). This association remained significant when considering recessive (OR = 0.548, 95% CI 0.376-0.800; P = 0.002) and additive (OR = 0.527, 95% CI 0.343-0.812; P = 0.004) inheritance models (Table 2).

The frequency of the T allele of the rs4324901 SNP in *ANGPT-1* was higher in controls compared to cases (36% *vs*. 31%; P = 0.026). Genotype frequency of this SNP did not differ significantly between groups (P = 0.064). When considering the dominant model, the T allele was significantly associated with protection against DR (P = 0.023), which was maintained after adjustment for age, HbA1c, gender, and presence of hypertension (OR = 0.701, 95% CI 0.510-0.963; P = 0.028). No association was found when considering the additive and recessive models (Table 2).

The frequency of the A allele of the *ANGPT-1* rs2507800 SNP was 37% in controls and 35% in cases with DR (P = 0.421). Genotype frequencies of this SNP did not differ significantly between groups (P = 0.656) or when considering different inheritance models (all P values > 0.050). Furthermore, adjustments for age, HbA1c, gender, and presence of hypertension did not change the lack of association between the rs2507800 SNP and DR (Table 2).

Moreover, when we compared control patients to patients with PDR, the *TIE2* rs639225 G/G and *TIE2* rs638203 G/G genotypes conferred protection against PDR (OR = 0.573; 95% CI 0.377-0.872; P = 0.012 and OR = 0.512; 95% CI 0.335-0.783; P = 0.003, respectively, for the recessive model). No association was found between the rs4324901 and rs2807800 SNPs in *ANGPT-1* and PDR (P = 0.279 and P = 0.338, respectively). Additionally, when comparing control patients to patients with NPDR, the presence of the T allele of the *ANGPT-1* rs4324901 SNP was associated with protection against NPDR (OR = 0.698, 95% CI 0.512-0.953; P = 0.029). No association was found between the *ANGPT-1* rs2807800 SNP (P = 0.915) and the *TIE2* rs639225 and rs638203 SNPs (P = 0.780 and P = 0.462, respectively) and NPDR.

### Haplotype distributions

The *TIE2* rs639225 SNP is in almost complete LD with the *TIE2* rs638203 SNP (|D’| = 0.971, r^2^ = 0.905) in our population. For this reason, we did not proceed with the haplotype analysis between these two SNPs. In contrast, the *ANGPT-1* rs4324901 SNP presented a weak LD with the *ANGPT-1* rs2507800 SNP (|D’| = 0.531, r^2^ = 0.258) in our population. Four haplotypes comprising the *ANGPT-1* rs4324901 and rs2507800 SNPs were inferred in both groups: T/G (51.8%), T/T (11.6%), A/G (14.7%), and A/T (21.9%). Their distributions did not differ significantly between groups (P = 0.080). However, after comparing patients carrying 0 to 2 minor alleles to patients with 3 or 4 minor alleles in the haplotypes (Table 2), the presence of ≥ 3 minor alleles in the *ANGPT-1* haplotypes seemed to be associated with protection against DR (20.8% in controls *vs*. 15.4% in cases; P = 0.054). This difference was significant after adjustment for age, HbA1c, gender, and presence of hypertension (OR = 0.602 95% CI 0.392-0.925; P = 0.021; Table 2).

### Association between SNPs in the *TIE2* and *ANGPT-1* genes and DKD

In order to evaluate whether the analyzed SNPs could also be associated with DKD in our population, we categorized our sample according to the presence of this complication: 349 patients without DKD *vs*. 761 patients with this complication. Frequencies of the *TIE2* rs639225, *ANGPT-1* rs4324901, and *ANGPT-1* rs2507800 SNPs did not differ between patients with or without DKD (Supplementary Table 1). However, the frequency of the *TIE2* rs638203 G/G genotype was lower in patients with T2DM and DKD compared to patients without this complication (19.3% *vs*. 24.9%, OR = 0.721, 95% CI 0.533-0.975; P = 0.040 for the recessive model), but this association was not independent of DR presence, age, gender, HbA1c, and hypertension in the logistic regression analysis (OR = 0.839, 95% CI 0.574-1.226; P = 0.365).

## DISCUSSION

The ANGPT family has been reported as being involved in DR pathogenesis as a mediator of the permeability of the blood-retinal barrier and a regulator of pericyte function, angiogenesis, and apoptosis (15, 36). Thus, considering that ANGPT plays an important role in DR, the present study investigated the association of four SNPs in the *TIE2* and *ANGPT-1* genes with DR in patients with T2DM. Our results show, for the first time, an association between the *TIE2* rs638203 G/G and *TIE2* rs639225 G/G genotypes and protection against DR. Moreover, the presence of the T allele of the *ANGPT-1* rs4324901 SNP and the presence of ≥ 3 minor alleles in the *ANGPT-1* haplotypes conferred protection against DR.

TIE2, also known as TEK, comprises immunoglobulin-like domains, epidermal growth factor-like domains, and fibronectin type III domains (15). After activation, TIE2 shows strong kinase activity and becomes phosphorylated on several cytoplasmic tyrosine residues, resulting in downstream activation of some pathways, such as PI3-kinase/protein kinase B (AKT) and extracellular signal regulated kinase (ERK) pathways (37). The activation of these pathways inhibits de novo blood vessel growth and vascular hyperpermeability (37), key processes in DR pathogenesis.

To our knowledge, no other study so far has investigated the frequencies of these two *TIE2* SNPs in patients with DR. The rs638203 and rs639225 SNPs were previously associated with risk of vascular malformations (38), and the rs639225 SNP was associated with a baseline peritoneal transport property (34). Few studies have reported TIE2 levels in patients with DM and DR (16, 39). Khalaf and cols. (16) reported that serum TIE2 levels were similar between patients with T2DM and PDR, NPDR, or without this complication. In accordance, another study found no difference in serum TIE2 levels in patients with T2DM with and without DR (39).

Although these results suggest that serum TIE2 levels are not associated with DR, TIE2 activation has been investigated as a treatment or prevention strategy of DR, since TIE2 activation increased EC survival and adhesion, as well as cell-cell junction integrity, thereby stabilizing the vasculature (40, 41). Thus, polymorphisms in *TIE2* gene associated with DR protection, such as the rs638203 SNP, could be involved with better TIE2 activation and, consequently, better function and stabilization of the vasculature, which is important in DR pathogenesis. However, to date, no study has evaluated the functional impact of the *TIE2* rs638203 SNP; hence, functional studies are necessary to better understand the involvement of *TIE2* SNPs in DR.

Regarding the *ANGPT-1* SNPs, we demonstrated an association of the presence of the T allele of the rs4324901 SNP and the presence of ≥ 3 minor alleles in *ANGPT-1* haplotypes with protection against DR. ANGPT-1 seems to be involved in DR pathogenesis since ANGPT-1 is reported to help delay diabetic complications owing to the restoration of microvascular function maintenance of quiescence in some adult stem cells [reviewed in (42)]. Accordingly, ANGPT-1 treatment protected retinal pericytes against apoptosis after 48 h of culture with tumor necrosis factor (TNF) or high glucose (36). Moreover, ANGPT-1 seems to improve the activation and migration of retinal pericytes during the establishment of new retinal vessels (36). Joussen and cols. (43) demonstrated that Angpt-1 protects the retinal vasculature of patients with DM against leukocyte-mediated EC injury and death and suppresses diabetic blood-retinal barrier breakdown and vascular endothelial growth factor (Vegf) and intercellular adhesion molecule 1 (Icam-1) expression in mice.

Expression levels of ANGPT-1 were also investigated in patients with DM (16,39,44). Serum ANGPT-1 levels in T2DM patients without DR and those of patients with T2DM and PDR were similar; however, patients with NPDR had higher ANGPT-1 levels compared to PDR and T2DM control groups (39). Furthermore, Khalaf and cols. (16) observed that serum ANGPT-1 levels were higher in patients with NPDR *vs*. DM control patients with DM without this complication, but no difference was found between patients with PDR and controls. These authors suggested that the increased expression of ANGPT-1 in patients with DR occurs early in the development of this complication as a compensatory mechanism to help cellular repair and preserve the integrity of ECs (16). Accordingly, when we stratified patients according to DR severity, the *ANGPT-1* rs4324901 T allele was associated with protection against NPDR but not PDR. Thus, we hypothesized that the T allele might increase *ANGPT-1* expression, thus conferring protection against the development of NDPR. Functional studies are also needed to confirm this hypothesis.

*ANGPT-1* expression seems to be altered by the rs2507800 A/A genotype. Chen and cols. (44) demonstrated that the A allele of the rs2507800 SNP suppressed *ANGPT-1* translation by facilitating miR-211 binding, which was not observed for the T allele. Accordingly, individuals carrying the T/T genotype had higher plasma ANGPT-1 levels than those with the A allele (44). Even though the rs2507800 SNP seems to alter ANGPT-1 levels, we did not demonstrate an individual association between this SNP and DR in the present study. Of note, no previous case-control study evaluated the association of *ANGPT-1* SNPs with DR.

The present study has a few limitations. First, we cannot rule out the possibility of population stratification bias when analyzing our samples. Both minor alleles of the *TIE2* SNPs did not differ significantly between white and non-white patients (all P values > 0.700). Regarding *ANGPT-1* SNPs, although their frequencies differed between white and non-white participants (P = 0.0001), when analyzing white and non-white patients separately, the T allele of the rs4324901 SNP remained increased in controls without DR in comparison to patients with DR in both ethnic groups. Second, we cannot fully exclude the possibility of a type II error when analyzing associations between the rs2507800 SNP in *ANGPT-1* and DR. Although we had more than 80% power (α = 0.050) to detect an OR ≤ 0.4 for DR protection and the sample sizes were in agreement to those calculated, we cannot exclude the possibility that this SNP would be associated with DR with a lower OR.

In conclusion, we demonstrated, for the first time, an association of rs638203 and rs639225 SNPs in *TIE2* and of rs4324901 SNP in *ANGPT-1* with protection against DR in a Brazilian population. These results are biologically plausible considering the involvement of TIE2 and ANGPT-1 in key pathways related to DR pathogenesis. No functional study has been published on the impact of these SNPs on *TIE2* and *ANGPT-1* expression, thus analyses are still required to clarify how they influence the expression of their respective genes as well as DR pathogenesis. Nevertheless, the present data contribute to the identification of new genetic markers of DR protection. Additional studies are needed to confirm the associations of these SNPs with DR in other populations and also in patients with T1DM.

## Supplementary Table 1 Genotype and allele frequencies of SNPs in *TIE2* and *ANGPT-1* genes in T2DM patients with and without DKD

**Table:**

	T2DM patients without DKD	DKD patients	Unadjusted P*	Adjusted OR (95% IC) /† P
**rs639225 – *TIE2***	337	742		
**Genotype**				
A/A	114 (33.9)	263 (35.4)	0.224	1
A/G	140 (41.5)	331 (44.7)		1.081 (0.756-1.546)/0.669
G/G	83 (24.6)	148 (19.9)		0.976 (0.633-1.504)/0.912
**Allele**				
A	0.55	0.58	0.186	-
G	0.45	0.42		
**Recessive model**				
A/A + A/G	254 (75.4)	594 (80.1)	0.097	1
G/G	83 (24.6)	148 (19.9)		0.935 (0.63-1.373)/0.730
**Additive model**				
A/A	114 (57.9)	263 (64.0)	0.172	1
G/G	83 (42.1)	148 (36.0)		0.939 (0.609-1.446)/0.774
**Dominant model**				
A/A	114 (33.8)	263 (35.4)	0.655	1
A/G + G/G	223 (66.2)	479 (64.6)		1.046 (0.752-1.454)/0.791
**rs638203 – *TIE2***	349	761		
**Genotype**				
A/A	119 (34.1)	264 (34.7)	0.088	1
A/G	143 (41.0)	350 (46.0)		1.159 (0.814-1.650)/0.412
G/G	87 (24.9)	147 (19.3)		0.912 (0.595-1.398)/0.672
**Allele**				
A	0.55	0.58	0.185	-
G	0.45	0.42		
**Recessive model**				
A/A + A/G	262 (75.1)	614 (80.7)	0.040	1
G/G	87 (24.9)	147 (19.3)		0.839 (0.574-1.226)/0.365
**Additive model**				
A/A	119 (57.8)	264 (64.2)	0.141	1
G/G	87 (42.2)	147 (35.8)		0.868 (0.566-1.332)/0.518
**Dominant model**				
A/A	119 (34.1)	264 (34.7)	0.900	1
A/G + G/G	230 (65.9)	497 (65.3)		1.073 (0.775-1.487)/0.671
**rs4324901 – *ANGPT-1***	331	726		
**Genotype**				
G/G	151 (45.6)	353 (48.6)	0.645	1
G/T	133 (40.2)	279 (38.5)		0.897 (0.634-1.268)/0.539
T/T	47 (14.2)	94 (12.9)		1.289 (0.790-2.103)/0.309
**Allele**				
G	0.66	0.68	0.115	-
T	0.34	0.32		
**Recessive model**				
G/G + G/T	284 (85.8)	632 (87.1)	0.647	1
T/T	47 (14.2)	94 (12.9)		1.355 (0.853-2.152)/0.198
**Additive model**				
G/G	151 (76.3)	353 (79.0)	0.506	1
T/T	47 (23.7)	94 (21.0)		1.328 (0.807-2.184)/0.264
**Dominant model**				
G/G	151 (45.6)	353 (48.6)	0.401	1
G/T + T/T	180 (54.4)	373 (51.4)		0.990 (0.718-1.364)/0.951
rs2507800 – ***ANGPT-1***	335	739		
**Genotype**			
T/T	150 (44.8)	313 (42.4)	0.270	1
T/A	129 (38.5)	321 (43.4)		1.256 (0.888-1.776)/0.197
A/A	56 (16.7)	105 (14.2)		1.231 (0.765-1.981)/0.391
**Allele**				
T	0.64	0.64	0.976	-
A	0.36	0.36		
**Recessive model**				
T/T + T/A	279 (83.3)	634 (85.8)	0.330	1
A/A	56 (16.7)	105 (14.2)		1.103 (0.706-1.723)/0.666
**Additive model**				
T/T	150 (72.8)	313 (74.9)	0.648	1
A/A	56 (27.2)	105 (25.1)		1.269 (0.780-2.063)/0.337
**Dominant model**				
T/T	150 (44.8)	313 (42.4)	0.499	1
T/A + A/A	185 (55.2)	426 (57.6)		1.249 (0.907-1.722)/0.174

Data are shown as number (%) or proportion.

* P-values were calculated using *x*^2^ tests.

† P-values and OR (95% CI) obtained using logistic regression analyses adjusting for age, gender, HbA1c, presence of hypertension and diabetic retinopathy.
